# Case Report: Where is the boundary between autosomal recessive early-onset Parkinson’s disease and dystonia-parkinsonism: a case of PLA2G6-associated neurodegeneration

**DOI:** 10.3389/fnhum.2026.1772073

**Published:** 2026-05-04

**Authors:** Xiao-Tian Li, Cheng-Xiang Yuan, Zhao-Qi Lv, Hui Hu, Xiao-Jie Zhang, Meng-Qian Ye, Jia-Xin Chen, Nuo Chen, Yan Li, Xiong Zhang, Jian-Yong Wang

**Affiliations:** 1 Department of Neurology and Institute of Geriatric Neurology, The Second Affiliated Hospital and Yuying Children’s Hospital, Wenzhou Medical University, Wenzhou, Zhejiang, China; 2 Wenzhou Key Laboratory of Neurogenetics, Wenzhou, Zhejiang, China

**Keywords:** autosomal recessive early-onset Parkinson’s disease, dystonia-parkinsonism, genetics, parkinsonism, PLA2G6

## Abstract

**Background:**

Mutations in the *PLA2G6* gene cause a spectrum of neurodegenerative disorders, with autosomal recessive early-onset Parkinson’s disease (AREP) and dystonia-parkinsonism (DP) representing the two primary subtypes of adult-onset PLA2G6-associated neurodegeneration (PLAN).

**Case presentation:**

We report a Chinese female patient with parkinsonism caused by compound heterozygous mutations in the *PLA2G6* gene. Both variants she carried—c.313G > T (p. D105Y) and c.23 T > A (p. V8D)—are novel and have not been previously reported. The patient presented with prominent parkinsonism in the upper body that responded well to dopaminergic therapy, while the lower limbs exhibited combined dystonia and a scissoring gait with poor response to dopaminergic medication. Based on the distinctive features of our case and literature review, we suggest that the traditional AREP/DP dichotomy may not fully capture the phenotypic complexity observed in *PLA2G6*-associated parkinsonism.

**Conclusion:**

This case highlights the clinical heterogeneity of PLAN and expands its genotypic spectrum with two novel mutations, suggesting that *PLA2G6*-related disorders may present with overlapping features of AREP and DP within the same individual.

## Introduction

Parkinson’s disease (PD) exhibits high clinical heterogeneity and is often accompanied by atypical symptoms. This poses significant challenges for the patient’s diagnosis and treatment. Meanwhile, several gene mutations have been identified as causes of PD, and patients with these mutations often present with atypical features (Blauwendraat et al., 2020). These atypical features include early disease onset, concomitant dementia, and poor response to medication.

The *PLA2G6* (known as *PARK14*) gene encodes calcium-independent phospholipase A2β (iPLA2β), which plays a critical role in membrane homeostasis and facilitating intracellular signaling (Malley et al., 2018). Mutations in the *PLA2G6* gene cause a spectrum of neurological disorders. The clinical presentation may include parkinsonism, dystonia, gait abnormalities, autonomic dysfunction, cognitive impairment, and other symptoms (Magrinelli et al., 2022). Adult-onset *PLA2G6*-associated neurodegeneration (PLAN) includes autosomal recessive early-onset PD (AREP) and dystonia-parkinsonism (DP). Currently, several studies have reported parkinsonism associated with *PLA2G6* gene mutations. The clinical phenotypes of these cases vary widely and appear to follow no discernible pattern (Magrinelli et al., 2022; Gao et al., 2022; Paisan-Ruiz et al., 2009; Chu et al., 2020; Bower et al., 2011).

In this study, we report a case of parkinsonism caused by compound heterozygous mutations in the *PLA2G6* gene. This patient exhibited a marked phenotypic divergence between the upper and lower body. Furthermore, both mutation sites are novel and have not been previously reported.

## Case presentation

The patient was a Chinese female who began experiencing difficulty walking at the age of 55. Her medical history includes hypertension and type 2 diabetes, both well-controlled on oral medication. She had no history of head trauma, encephalitis, or toxin exposure. Her parents were non-consanguineous. Her father was deceased, and no medical records were available for review. Her mother and son were healthy and asymptomatic. One of her younger brothers was diagnosed with PD at the age of 30. No other family members across three generations had a history of neurological disorders, including parkinsonism, dystonia, or cognitive impairment. With the progression of the disease, she gradually developed bradykinesia, rigidity, difficulty turning over, unsteady gait, and recurrent falls. At the age of 57, a DAT-scan revealed reduced dopamine transporter levels in the bilateral putamen, with more significant reduction on the left side. Based on this, she was diagnosed with PD and prescribed benserazide/levodopa 25/100 mg three times daily, pramipexole 0.5 mg three times daily, and levodopa/carbidopa/entacapone 100/25/200 mg once daily. Her bradykinesia and rigidity improved, but the gait disturbance persisted. At the age of 58, the patient was admitted to the Second Affiliated Hospital of Wenzhou Medical University due to deteriorating gait impairment.

Neurological examinations revealed that the patient was alert but with reduced verbal fluency. No abnormalities were detected on cranial nerve examination. Muscle strength and sensory function were intact in all four limbs. She had increased muscle tone and bradykinesia in all four limbs, both of which were more pronounced on the right side. The patient had brisk reflexes (+++) in both upper limbs, diminished reflexes (+) in the lower limbs, and an absent Babinski sign. Dystonic involuntary movements were present in her left lower limb (Supplementary Video 1). Examination of her gait revealed a prominent scissoring component with associated hip weakness (Supplementary Video 2). Other neurological signs were unremarkable.

**Figure 1:**
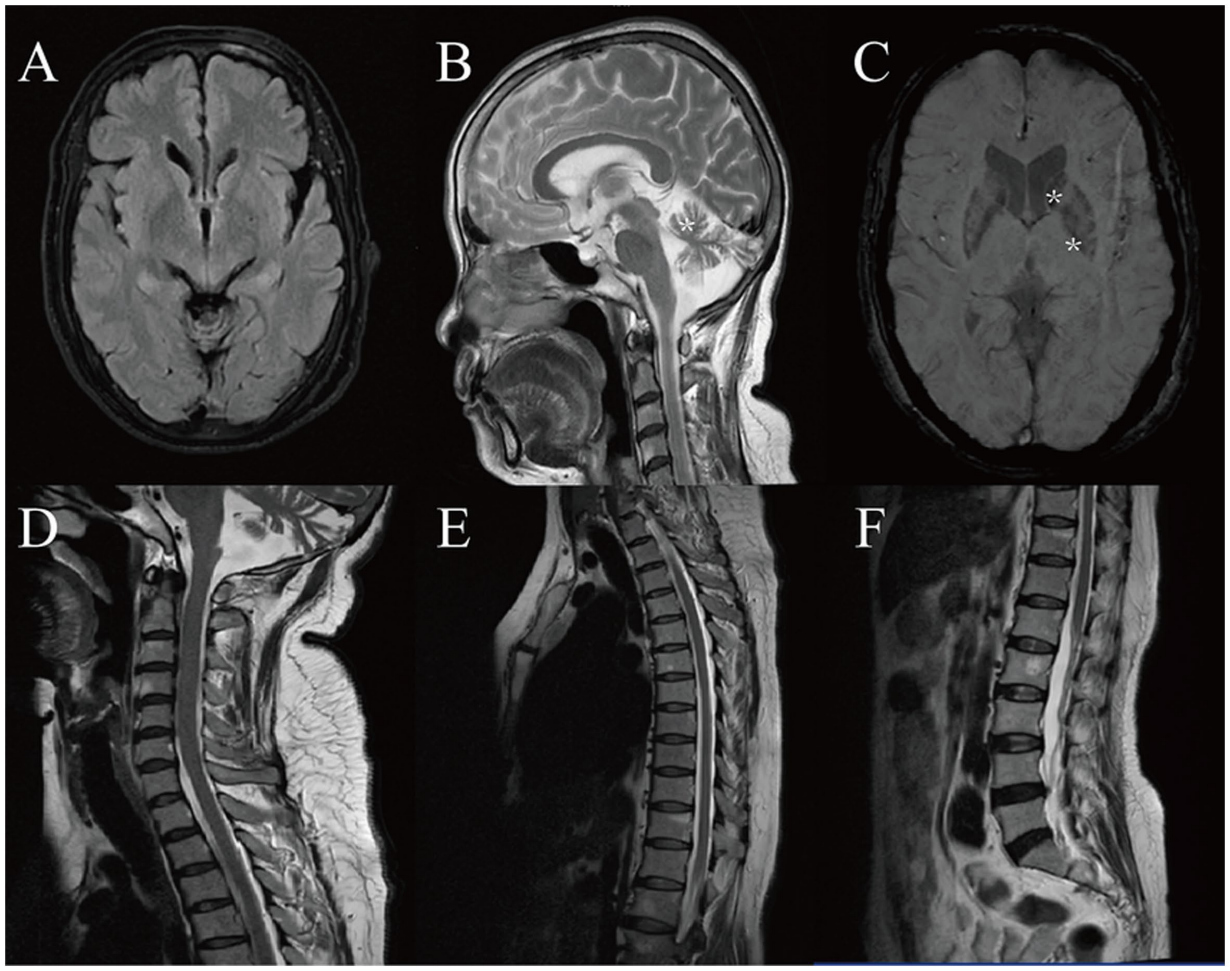
Brain and spine magnetic resonance imaging (MRI) of the patient. **(A)** Fluid attenuated inversion recovery image, showing mild global cerebral atrophy. **(B)** Sagittal view of T2-weighted image, showing marked cerebellar atrophy (*). **(C)** Susceptibility-weighted image, showing iron deposition in the caudate nucleus and globus pallidus (*). **(D)** T2-weighted cervical spine MRI. **(E)** T2-weighted thoracic spine MRI. **(F)** T2-weighted lumbar spine MRI.

The laboratory parameters including complete blood count, C-reactive protein, plasma lactate, coagulation indices, ceruloplasmin, liver, kidney, and thyroid levels were within normal range. The brain magnetic resonance imaging (MRI) revealed global cerebral atrophy (Figure 1A), particularly in the cerebellum (Figure 1B). Susceptibility-weighted imaging revealed iron deposition in the patient’s caudate nucleus and globus pallidus (Figure 1C). No significant abnormal signals were detected on her cervical, thoracic, and lumbar MRI scans (Figures 1D–F). No abnormalities were detected on electrocardiogram, electromyogram, or electroencephalogram. Her Unified Parkinson’s Disease Rating Scale total score was 79, with 54 points in motor examination (part III). The Mini-Mental State Examination score was 12 and the Montreal Cognitive Assessment score was 9.

**Figure 2:**
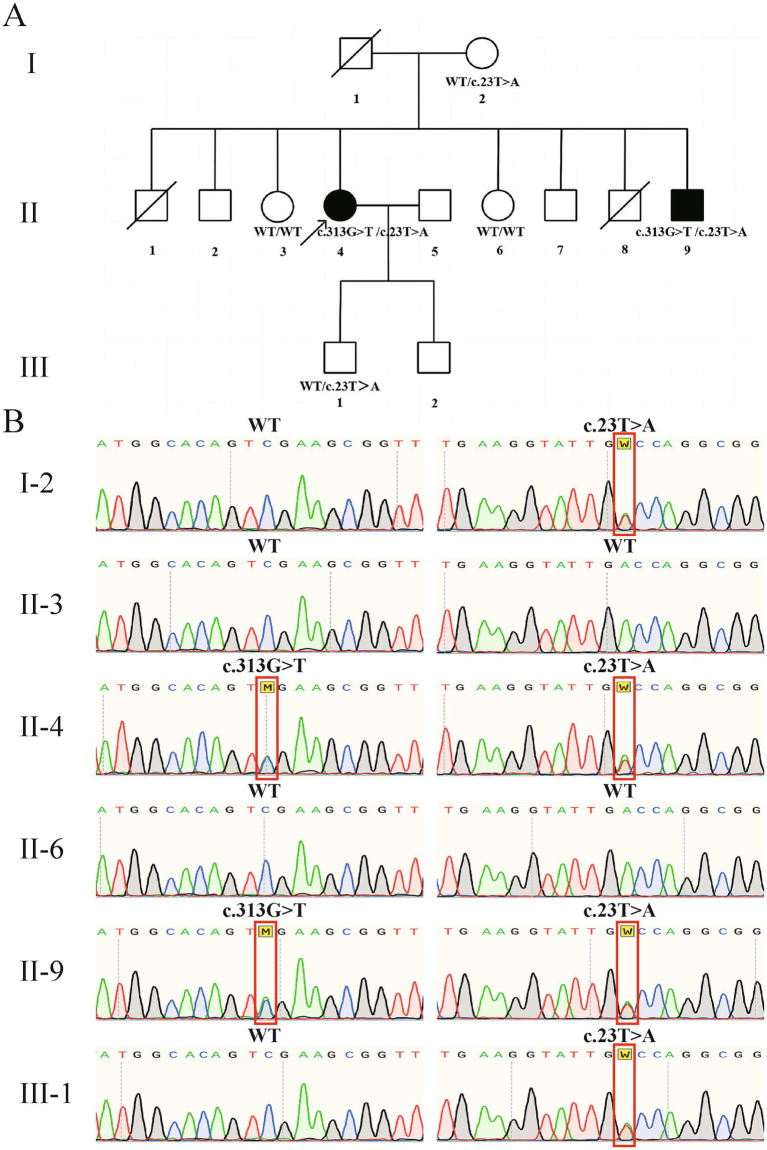
Pedigree and Sanger sequencing. **(A)** Pedigree of the family. The pedigree spans three generations (I–III). Squares represent males, circles represent females, and the arrow indicates the proband (II-4). Filled symbols denote clinically affected individuals presenting with parkinsonism, while open symbols represent asymptomatic individuals. A diagonal line through a symbol indicates a deceased family member. Genetic testing results for the two identified *PLA2G6* mutations are shown below each available individual. **(B)** Sanger sequencing of the patient and her family members. Left panel shows the c.313G > T (p. D105Y) mutation in exon 6. Right panel shows the c.23 T > A (p. V8D) mutation in exon 2. Red boxes indicate the nucleotide changes. WT, wild type.

The initial diagnosis of idiopathic PD was revised based on atypical features (early onset, poor gait response to levodopa, family history, and cerebellum atrophy), which led to genetic testing. Exome sequencing identified that the patient carries compound heterozygous mutations in *PLA2G6*, specifically c.313G > T (p. D105Y) and c.23 T > A (p. V8D). We performed Sanger sequencing on peripheral blood DNA from some members of the patient’s family. The results confirmed that both the patient and one of her brothers, who was diagnosed with “PD,” carry the compound heterozygous mutations in the *PLA2G6* gene. Unfortunately, due to insufficient clinical information, we are unable to determine whether her brother exhibits atypical features similar to hers. Her mother and son both carried the c.23 T > A (p. V8D) variant but remained asymptomatic, which is consistent with an autosomal recessive inheritance pattern (Figures 2A,B). Both mutations are missense and are not documented in dbSNP^1^. The c.313G > T (p. D105Y) mutation is located in exon 6 of the *PLA2G6* gene, was predicted as “possibly damaging” by PolyPhen-2^2^, and was categorized as “affect protein function” by SIFT^3^. The c.23 T > A (p. V8D) mutation is located in exon 2 of the *PLA2G6* gene, was predicted as “probably damaging” by PolyPhen-2, and was categorized as “affect protein function” by SIFT.

Based on the patient’s clinical presentation, imaging findings, family history, and genetic test results, we diagnosed her with PLAN (Magrinelli et al., 2022), presenting with mixed features of parkinsonism and dystonia. The patient was treated with levodopa/carbidopa/entacapone 100/25/200 mg three times daily for her parkinsonism. Her bradykinesia and muscle rigidity in the upper limbs improved significantly, but her gait disturbance and left lower limb dystonia persisted. We recommended that the patient persist with rehabilitation training to improve her gait. The patient is currently undergoing regular follow-ups at our movement disorders clinic. At her most recent visit 6 months after admission, upper limb symptoms remained well-controlled on current medication, while lower limb dystonia and gait impairment persisted with minimal change. No adverse effects from medication have been observed. She has adapted to her condition and reports satisfaction with the current treatment and supportive care. Adherence to rehabilitation exercises is good, though objective gait improvement remains limited.

## Discussion

PLAN represents a series of neurological disorders with marked heterogeneity in mutation sites, clinical presentations, and age of onset. Herein, we report a case of parkinsonism caused by compound heterozygous mutations (c.313G > T [p. D105Y] and c.23 T > A [p. V8D]) in the *PLA2G6* gene. The patient exhibited significant divergence in clinical manifestations between the upper and lower body, with levodopa-responsive parkinsonism in the upper limbs and levodopa-resistant dystonia with scissoring gait in the lower limbs.

Early-onset PD (EOPD) refers to patients with PD with onset before the age of 50. It is often accompanied by distinctive symptoms, such as psychiatric symptoms and pain (Poplawska-Domaszewicz et al., 2024). Genetic mutations are an important cause of EOPD, and PLA2G6 is one of the genes involved. The *PLA2G6* gene, located on chromosome 22, encodes iPLA2β, whose functions include regulating cell proliferation, maintaining membrane stability, and controlling apoptosis (Malley et al., 2018; Ackermann et al., 1994). Mutations in the *PLA2G6* gene lead to a spectrum of disorders—including infantile neuroaxonal dystrophy, atypical infantile neuroaxonal dystrophy, DP, and AREP—which exhibit a wide range in the age of onset. DP and AREP are two common subtypes of adult-onset PLAN.

It is generally accepted that patients with AREP primarily present with parkinsonism as the core manifestation and typically show a favorable response to dopaminergic therapy, whereas those with DP exhibit prominent dystonia and generally demonstrate a poor response to dopaminergic medications. Related cases are often conventionally categorized as either AREP or DP in clinical reports (Gao et al., 2022; Chu et al., 2020; Karkheiran et al., 2015; Xie et al., 2015; Bower et al., 2011). To critically evaluate the validity of the AREP/DP dichotomy, we systematically reviewed previously reported PLA2G6 genotypes and their associated phenotypes (Table 1). The reported mutation sites are widely distributed across the gene, and most of them are compound heterozygous mutations. The genotype–phenotype correlation appears to be poorly defined. For instance, the homozygous c.991G > T mutation manifests as AREP, whereas compound heterozygous mutations harboring c.991G > T may present as either AREP or DP. Homozygous mutations c.2239C > T and c.2222G > A can both lead to either AREP or DP (Paisan-Ruiz et al., 2009; Giri et al., 2016; Demir Unal et al., 2022; Virmani et al., 2014; Bohlega et al., 2016). Unlinked modifier genes, epigenetic changes, and environmental factors are important contributors to the marked clinical heterogeneity (Cooper et al., 2013). Our patient exhibited a combination of features that do not fit neatly into either category: classic drug-responsive parkinsonism in the upper body and drug-resistant dystonia with scissoring gait in the lower limbs. Several interpretations of this observation are possible. First, the patient may represent a genuine phenotypic continuum between AREP and DP, as previously suggested (Gao et al., 2022). In this scenario, the distinction between the two syndromes is blurred throughout the individual, without a clear regional separation. Second, the patient might simultaneously manifest two distinct phenotypes—AREP and DP—each attributable to the same mutation but localized to different body regions (e.g., AREP features in the upper body and DP features in the lower body). Third, the apparent divergence between upper and lower body manifestations could instead reflect regional heterogeneity in the expression of a single underlying disease process, without necessarily meeting the diagnostic criteria for either AREP or DP as separate entities. In this scenario, the patient has a unified pathology, and the regional differences do not represent a true overlap of two distinct syndromes. Our single case does not allow definitive discrimination among these possibilities. Two systematic reviews of this disease also documented overlapping features between the two phenotypes, despite the authors’ employment of precise nomenclature in the original case reports (Karkheiran et al., 2015; Chu et al., 2020). Certainly, some reported cases have not made a deliberate distinction between the specific subtypes (Klein et al., 2016). A study analyzing the symptom spectrum of patients with PLAN found diverse combinations of clinical manifestations, with parkinsonism present in nearly all affected individuals (Magrinelli et al., 2022). Considering that patients may exhibit diverse symptoms across different disease stages and, as illustrated by our case, distinct core manifestations in different body regions, we propose that using the term “PLA2G6-Associated Parkinsonism” is more accurate, as it represents a spectrum of phenotypes caused by mutations in the same gene.

**Table 1:** The spectrum of *PLA2G6* genotypes and their associated clinical phenotypes.

References	Genotypes	Phenotypes
Yoshino et al., 2010	c.216C > A/c.1904G > A	AREP
	c.1354C > T/c.1904G > A	
Klein et al., 2016	c.610-1G > T/c.1627C > T	
Wirth et al., 2017	c.109C > T/c.2321G > T	
	c.758G > T/c.2341G > A	
Chen et al., 2018	c.668C > T/c.1915G > A	
Shen et al., 2019	c.991G > T/c.1472 + 1G > A	
Gao et al., 2022	c.991G > T/c.1427 + 1G > A	
Cai et al., 2024	c.991G > T/c.1454G > A	
Kauther et al., 2011	c.2339A > G/c.2339A > G	
	c.2341G > A/c.2341G > A	
Shi et al., 2011	c.991G > T/c.991G > T	
Xie et al., 2015	c.991G > T/c.991G > T	
Chu et al., 2020	c.991G > T/c.991G > T	
Huang et al., 2024	c.1898C > T/c.1898C > T	
Bower et al., 2011	c.4C > A/Del Ex3	DP
Huang et al., 2018	c.991G > T/c.1077G > A	
Chu et al., 2020	c.991G > T/c.1077G > A	
Kim et al., 2015	c.1039G > A/c.1670C > T	
Chen et al., 2018	c.991G > T/c.1982C > T	
Hua et al., 2022	c.991G > T/c.1634A > G	
Sina et al., 2009	c.1894C > T/c.1894C > T	
Agarwal et al., 2012	c.238G > A/c.238G > A	
Malaguti et al., 2015	c.1547C > T/c.1547C > T	
Rohani et al., 2018	c.2041delC/c.2041delC	
Yamashita et al., 2017	c.1495G > A/c.1495G > A	
Kamel et al., 2019	c.2215G > C/c.2215G > C	
Vela-Desojo et al., 2022	c.1435C > G/c.1435C > G	
Paisan-Ruiz et al., 2009	c.2239C > T/c.2239C > T	AREP or DP
Giri et al., 2016	c.2239C > T/c.2239C > T	
Demir Unal et al., 2022	c.2239C > T/c.2239C > T	
Paisan-Ruiz et al., 2009	c.2222G > A/c.2222G > A	
Virmani et al., 2014	c.2222G > A/c.2222G > A	
Bohlega et al., 2016	c.2222G > A/c.2222G > A	

AREP, autosomal recessive early-onset Parkinson’s disease; DP, dystonia-parkinsonism.

Different manifestations of genetic diseases may coexist or exhibit temporal evolution, as previously reported (Zeng et al., 2025). In our patient, it was difficult to delineate the pattern of disease onset, as she already exhibited drug-responsive parkinsonism in the upper body and drug-resistant dystonia with a scissoring gait in the lower limbs at the initial presentation. In such patients, regular follow-up and careful documentation of clinical features are of great importance.

To date, dozens of pathogenic variants in the *PLA2G6* gene have been reported. To our knowledge, both the c.313G > T (p. D105Y) and c.23 T > A (p. V8D) variants carried by our patient have not been previously reported. Both the two mutations were predicted by PolyPhen-2 and SIFT to affect protein function and are likely pathogenic. More importantly, in the patient’s family, members carrying compound heterozygous mutations developed parkinsonism, while those with a single heterozygous variant remained asymptomatic. This observation is consistent with the pattern of autosomal recessive inheritance. Therefore, we conclude that both c.313G > T (p. D105Y) and c.23 T > A (p. V8D) are pathogenic variants.

To better integrate genotype and phenotype, we examined the specific mutation characteristics in relation to clinical variability. The p. V8D mutation resides in the N-terminal region, which is thought to influence membrane association and protein stability. The p. D105Y mutation lies within the ankyrin repeat domain, a region critical for protein–protein interactions and enzyme regulation, and known for phenotypic pleiotropy, as exemplified by the homozygous c.2222G > A mutation in the same domain, which has been reported to cause both DP and AREP (Paisan-Ruiz et al., 2009; Virmani et al., 2014; Bohlega et al., 2016). However, whether the combination of a N-terminal variant (p. V8D) and an ankyrin repeat domain variant (p. D105Y) accounts for the regional phenotypic divergence remains speculative and requires further functional studies and additional cases for validation.

Parkinsonism is highly heterogeneous, and genetic forms of PD represent an important and often underrecognized cause. Based on this case, we propose that several atypical clinical features—including concomitant dystonia, early onset, rapid progression, poor response to dopaminergic therapy, brain atrophy, intracerebral iron deposition, and a positive family history—may serve as indicators for genetic testing. Furthermore, for patients with suboptimal responses to pharmacological treatment, active rehabilitation training may provide additional benefit.

In summary, we report two novel *PLA2G6* mutations in a patient with striking regional phenotypic variability—levodopa-responsive parkinsonism in the upper body and levodopa-resistant dystonia in the lower limbs. This case challenges the strict dichotomy between AREP and DP and suggests that “PLA2G6-Associated Parkinsonism” is a more appropriate term. Our findings expand the genotypic and phenotypic spectrum of PLAN and highlight the importance of considering this diagnosis in patients with early-onset, atypical parkinsonism.

## Data Availability

The original contributions presented in the study are included in the article/Supplementary material, further inquiries can be directed to the corresponding authors.

## Footnotes

1
https://www.ncbi.nlm.nih.gov/snp/

2
http://genetics.bwh.harvard.edu/pph2/

3
https://sift.bii.a-star.edu.sg/
