# Supplementary material for: Case Report: Where is the boundary between autosomal recessive early-onset Parkinson’s disease and dystonia-parkinsonism: a case of PLA2G6-associated neurodegeneration
Source: Front Hum Neurosci. 2026 May 4;20:1772073. doi: 10.3389/fnhum.2026.1772073 (PMC13180865; doi:10.3389/fnhum.2026.1772073)
Supplement: Supplementary file 1 [file Data_Sheet_1.DOCX]

**Supplementary Video legends**

**Supplementary Video 1:** Dystonic involuntary movements were observed in her left lower limb.

**Supplementary Video 2:** The patient’s gait exhibited a scissoring pattern with associated hip muscle weakness.
